# Seven in every ten khat chewers in Gondar City had an intention to stop khat chewing: cross-sectional study using Transtheoretical Model

**DOI:** 10.1186/s12888-020-02984-4

**Published:** 2020-12-02

**Authors:** Asmamaw Adugna, Telake Azale, Simegnew Handebo

**Affiliations:** Department of Health Education and Behavioral Sciences, Institute of Public Health, College of Medicine and Health Sciences, University of Gondar, P.O. Box: 196, Gondar, Ethiopia

**Keywords:** Intention to quit, Khat, Trans theoretical model, Gondar, Ethiopia

## Abstract

**Background:**

Khat chewing practice is rapidly expanding worldwide and currently, an estimated over 10 million people chew khat daily. The transtheoretical model explains how behavior change occurs across the stages of change. So, this study aimed at assessing the intention to stop khat chewing and associated factors among khat chewers in Gondar City, northwest Ethiopia.

**Methods:**

Community-based cross-sectional study was conducted from March to April 2019 in Gondar City, northwest Ethiopia. Six hundred five khat chewers participated in the study from six randomly selected kebeles. The data were collected using structured and interviewer-administered questionnaire. Bivariate and multiple logistic regression models were fitted. Adjusted Odds ratio (AOR) with 95% confidence interval (CI) and *p*-values less than 0.05 were used to declare a significant association.

**Results:**

Of the study participants, 69.3% (95% CI: 65.8–72.9) had the intention to stop khat chewing within 6 months or before. The majority of the participants (55.4%) were at the contemplation stage. Higher self-reevaluation [AOR = 2.8, (95%CI: 1.6–5.0)], environmental reevaluation [AOR = 1.9, (95%CI: 1.1–3.3)] and social liberation [AOR = 1.8, (95% CI: 1.0–3.1)] were associated with intention to quit khat chewing. In addition, using additional substances daily, khat dependency, and early age initiation of khat chewing were associated with lower intention to quit khat chewing.

**Conclusion:**

The majority of the khat chewers had the intention to quit khat chewing. Increased self-reevaluation, environmental reevaluation, and social liberation process of changes were associated with enhanced intention of quitting khat chewing. Thus, stage-based interventions should be done to inspire khat chewers to realize their motivation of stopping khat chewing.

## Background

Khat (*Catha edulis*) is an evergreen plant that grows at high altitudes between 1500 and 2000 m above the sea level. It is commonly grown throughout the year in the Horn of African and Middle East countries. Khat leaves have been used as a stimulant for recreational purposes for centuries in the Horn of Africa and the Arabian Peninsula [1–7]. It also used for social and religious purposes [8]. Khat contains psychoactive ingredients which affect consciousness, behavior, mood, and thinking processes of people [9]. These active ingredients have the potential to lead physical and psychological dependence [2, 8–10]. However, chewers believed that it will keep them alert, attain greater concentration, boosts pleasure, and enhances motivation [10].

Currently, khat chewing is becoming a common practice among young segment of the population in Ethiopia. A meta-analysis of 24 studies on the prevalence of khat chewing revealed that 23.2% of university students chew khat [11]. According to the Ethiopian Demographic and Health Survey (EDHS) 2016 report, 27% of men and 12% of women ever chew khat and two in three of them chewed for 6 or more days in the last 30 days preceding the survey. The prevalence of khat chewing significantly varies among regions with the highest in Harari and lowest in Tigray, educational level and wealth status [12].

Chewing khat has multiple medical, sexual, economic and psychosocial problems. Studies revealed that habitual khat chewing results in cognitive impairment, learning problems, and behavioral abnormalities. It accounts for gastrointestinal tract problems like esophagitis, gastritis, and a delay in intestinal absorption. Moreover, khat chewing also causes dental, cardiovascular, and genitourinary problems [13–17]. Khat chewing has been found to affect the economy by decreasing the production as a result of tiredness and absenteeism. In some cases, workers go to lunch and engage in khat sessions, and may not return to work [18].

A qualitative study among former khat users identified numerous reasons that motivate khat chewers to make a decision on quitting khat use. The most common reasons were feeling of guilt for giving up prayers, feeling lost and neglecting family, accumulation of debts, work neglect and frequent absenteeism, and impaired health. Besides sexual and religious factors were the most pressing reasons for quitting [18]. The Transtheoretical Model (TTM) assumes that people do not change behaviors quickly and decisively; as well individuals who don’t have a plan will remain stuck in the early stages of behavior change. The change in behavior, especially habitual behavior, occurs continuously through a cyclic process [19].

Studies on an unassisted quitting attempt among daily khat consumers showed that although khat chewers have the motivation and desire to quit, they encountered difficulties in maintaining abstinence. An unaided quitting attempt has low success and withdrawal symptoms are the major barriers to quit khat chewing. The discomfort following discontinuation of khat chewing increase the risk of relapse. Treatment and behavioral intervention are crucial to assist khat chewers’ in quitting khat use [20, 21].

Empirical evidences suggest the TTM as the dominant model of health behavior change. The TTM enables to appreciate an individual’s readiness to act on a new healthy behavior and describe how people move through the stages of behavioral changes. The four main constructs of the model are: stages of change, processes of change, decisional balance, and self-efficacy. The model indicates that individuals who are trying to change their behavior pass thru a series of five exclusive stages of change, namely: Pre-contemplation, Contemplation, Preparation, Action and Maintenance. The movement through these stages occurs in cyclic patterns. People apply cognitive, affective, and evaluative processes of change to progress through these stages of change [22]. Ten processes of change classified in two categories facilitate the transition from one stage to the next. The experiential processes of change are used primarily for the early stage transitions and include: consciousness raising, dramatic relief, environmental reevaluation, social liberation, and self-reevaluation. The behavioral processes of changes used in the later stage transitions are: stimulus control, helping relationships, counter conditioning, reinforcement management and self-liberation. Each process of change intervenes uniquely at one transition [22].

The current study focused on assessing the intention to stop khat chewing among chewers using TTM. Intention to quit khat chewing is conceptualized as a readiness to engage in the quitting process [23]. Although using intention to predict behavior was debatable [24], evidences support that intention to engage in the behavior enable to predict whether the behavior will occur or not [23]. Intention has low impact on behavior when control over the behavior is lacking, social reaction exist, and conditions are conducive to habit formation [25]. Planning, maintaining self-efficacy, and control of action serves to mediate between early intention and behavior. The higher the intention, the more likely the behavior will be performed [24, 26]. Theory-based measurement of behavior changes is important to organize thinking about the health problem as well as the development and refinement of interventions. Despite numerous studies on the prevalence of khat use were conducted in Ethiopia, quitting and intention to quit khat chewing area was not well-investigated. Therefore, this study assessed the magnitude of intention to quit khat chewing and associated factors among chat chewers in Gondar City, northwest Ethiopia.

## Methods

### Study setting and design

A community-based cross-sectional study was conducted from March 15 to April 14, 2019, in Gondar, northwest Ethiopia. Gondar City is found in the central Gondar zone of Amhara regional state and located 727 km North West of Addis Ababa. There are 21 administrative kebeles with an estimated total population of 338,746 (160,522 males & 178,223 females) [27]. The city is found 2133 m above mean sea level with annual rainfall up to 1200 cubic millimeters. In the city, there are 160 licensed and functional khat chewing houses and shops [28].

### Study population and sample size

The study populations of this study were all khat chewers 18 and above years old and who lived in the city at least for 6 months. The sample size was determined by using the formula for the estimation of a single population proportion with an assumption of 95% confidence interval, 5% margin of error, and 50% of the expected proportion of khat chewer had an intention to quit khat chewing. To compensate for potential the non-response rate, 10% of the sample size was added. Then, the final sample size was 634. To recruit the study participants, first 6 kebeles were selected by a simple random sampling method. Then, all khat chewing houses and shops in the selected kebeles were identified and the sample size was proportionally allocated to each chewing houses and shops. Then, study participants were recruited randomly from each chewing house and shop until the sample size was met.

### Measures

The first three of stages changes in TTM (pre-contemplation, contemplation, and preparation) are relevant to measure intention. Pre-contemplates were chewers who were not seriously thinking about quitting khat chewing in the next 6 months. Contemplates were the chewers who reported that they were thinking about quitting in the next 6 months but not in the next 30 days. Whereas, khat chewers at preparatory stages were those who plan to quit in the next 30 days and made an attempt in the last year [22]. So, intention to stop khat chewing is measured as dichotomous variable “If the participant had a plan to stop khat chewing within 6 months or before ‘Yes’=1; Otherwise ‘No’ = 0” i.e. contemplation and preparation stage of change.

In addition to socio-demographic variable and chewing history, five processes of change of khat chewing: consciousness-raising, dramatic relief, environmental re-evaluation, social liberation, and self-reevaluation were assessed. Twenty Likert scale questions (1 = Strongly Disagree 2 = Disagree 3 = Neutral 4 = Agree 5 = Strongly Agree) were used to measure these processes of changes. The mean score of the five dimensions was re-coded as low (< 3), medium (3) and high (> 3) [29, 30].

Self-efficacy, the confidence that individuals can manage temptations for not chewing khat, was measured by 20 Likert scale questions (1 = Not Very Confident 2 = Not Confident 3 = Neutral 4 = Confident 5 = Very Confident) and categorized as having low, intermediate and high self-efficacy if they had a mean score of < 3, 3 and > 3 respectively. Decisional balance is the balance between the perceived advantages of chewing khat and the perceived disadvantages of chewing khat was measured by 18 Likert scale items (9 items for each) (1 = Strongly Disagree 2 = Disagree 3 = Neutral 4 = Agree 5 = Strongly agree) and the total score was re-coded as positive, undecided and negative based on the result of total pros minus total cons score of chewing [29, 30].

Self-reported khat dependence, level of khat dependence, was measured by 10 Likert items questions (1 = Strongly Disagree 2 = Disagree 3 = Neutral 4 = Agree 5 = Strongly agree) and the total score was recoded as low, medium and high dependence for a total score of < 3, 3 and > 3 respectively [29, 30].

### Data collection procedures

Interview administered, structured and translated questionnaire adapted from the different studies was used to collect the data [29–32]. The questionnaire was also pretested on 5% (32 participants) in Teda town. The reliability test for Cronbach’s alpha of the consciousness raising (0.80), dramatic relief (0.78), environmental reevaluation (0.85), self-reevaluation (0.86), social liberation (0.74), self-efficacy (0.87), decisional balance (pros of khat chewing) (0.82), decisional balance (cons of khat chewing (0.85), and khat dependency scale (0.74) subscales were maintained. With the supervision of two supervisors and principal investigator, questionnaires were filled by 6 BSc degree holder Nurses. Intensive training was given to the data collectors and supervisors on the research tool, the purpose of the study, interviewing techniques and handling of ethical issues by the primary investigator.

### Data processing and analysis

Data were entered into the EPI - info version 7 statistical software and exported to SPSS (Statistical Package for Social Science) version 20 for analysis. Descriptive statistics analysis was done to see the distribution of socio-demographic characteristics, process of changes, and intention to quit khat chewing. After the bivariable analysis was done, variables with *p*-values of < 0.2 were entered into a multiple logistic regression model to identify predictors associated with intention to quit khat chewing. These factors were expressed by AOR (Adjusted Odds Ratio) with 95% CI (Confidence Interval) and a probability value of type − 1 error less of less than 0.05 was considered statistically significant.

## Results

### Socio demographic characteristics

Of the 634 selected participants, 605 were successfully interviewed, with a response rate of 95.4%. Five hundred three (83.1%) were males. The median age of the respondents was 27 years (23 and 34 years were the 1st and 3rd quartiles respectively). With regard to educational status, 270 (44.6%) attended a diploma and above. The majority 384 (63.5%) of them were single. Above one in each four (26.9%) of the study participants had no job (See Table 1).

**Table 1:** Socio demographic characteristics of Khat chewers in Gondar city, north west Ethiopia; 2019 (*n* = 605)

Socio demographic characteristics	Frequency	Percent
Sex		
Male	503	83.1
Female	102	16.9
Age in years		
18–24	200	33.1
25–29	159	26.3
30–34	106	17.5
35 and above	140	23.5
Religion		
Christian	377	62.3
Muslim	191	31.6
Protestant	22	3.6
Other (Catholic, no religion)	15	2.5
Ethnicity		
Amhara	513	84.8
Tigrie	38	6.3
Oromo	37	6.1
Other (Kimant, Gurage, Sidama)	17	2.8
Educational status		
Can’t read & write	30	5.0
Read & write	28	4.6
Primary education	55	9.1
Secondary education	222	36.7
Diploma & above	270	44.6
Occupation		
Government employee	89	14.7
Merchant	132	21.8
Self-employee	93	15.4
Student	85	14.0
Job seeker	163	26.9
Other (driver, Guard)	43	7.1
Marital status		
Single	384	63.5
Married	174	28.8
Divorced	21	3.5
Other (separated, widowed)	26	4.1
Monthly income in birr		
< 2000	302	49.9
2000–3500	134	22.1
> 3500	169	28.0

### Khat dependency and chewing history

Of the total respondents, 263(43.5%) had high khat dependence and 310(51.2%) of the total respondents had a low dependency. The mean age for initiation of Khat chewing was 20.42 (SD ± 4.3) years. The mean duration of Khat chewing was 8.67 (SD ± 6.5) years. Two hundred thirty (38.0%) of the participants chewed khat for 4.39 (SD ± 2.6) hours per week. With regard to the purpose of chewing most of the participants, 317 (52.4%) chewed khat for recreation and 129 (21.3%) for reading. For about three-fourth 447 (73.9%) of the participants, khat chewing was a means of social interaction. Chewing was combined with cigarette smoking for 220 (36.4%) and alcohol drinking for 170 (28.1%) participants.

### Stages of change

Out of the total participants, 419 (69.3%) (95% CI: 65.8–72.9) have an intention to stop khat chewing at least within the next 6 months. The majority of the study participants 335 (55.4%) were on the contemplation stage. Whereas 84 (13.9%) were on the preparation stage and the rest 186 (30.7%) were on the pre-contemplation stage.

### Processes of change and self-efficacy

Nearly half of the participants had low consciousness-raising (48.3%) and high dramatic relief (46.8%) scores. The majority of them had high self-reevaluation (60.3%) social liberation (53.4%) and environmental reevaluation (62.5%) scores. The respondent’s self-efficacy to stop khat chewing revealed that half (50.9%) had low self-efficacy and 276 (45.6%) had high self-efficacy. Only 21(3.5%) of them had medium self-efficacy to stop khat chewing (See Table 2).

**Table 2:** Process of changes and self-efficacy of stopping khat chewing among khat chewers in Gondar city, northwest Ethiopia; 2019 (*n* = 605)

Variable	Frequency	Percent (%)
Consciousness raising		
Low	292	48.3
Medium	53	8.8
High	260	43.0
Dramatic relief		
Low	273	45.1
Medium	49	8.1
High	283	46.8
Self-reevaluation		
Low	206	34.0
Medium	34	5.6
High	365	60.3
Social liberation		
Low	215	35.5
Medium	67	11.1
High	323	53.4
Environmental reevaluation		
Low	190	31.4
Medium	37	6.1
High	378	62.5
Self-efficacy		
Low	308	50.9
Medium	21	3.5
High	276	45.6

### Decisional balance to stop khat chewing

This study found that for the majority (57.4%) of the respondent, the mean score of cons of khat chewing was higher than the pros score (negative decisional balance). On the other hand, for 38.3% of them, the mean score of pros of khat chewing was higher than the cons (positive decisional balance) and 26 (4.3%) of respondents were undecided.

### Factors associated with intention to stop khat chewing

As indicated in Table 3, in multiple logistic regression analysis age at initiation of khat chewing, frequency of additional drugs use, environmental reevaluation, self-reevaluation, and social liberation variables were significantly associated with intention to quit khat chewing. The odds of intention to quit khat chewing increased among adults who started chewing at an older age compared to those started chewing in early age. Adults who started chewing khat in the age group 16–20 years [AOR = 3.2, (95% CI; 1.5–6.9)], 21–25 years [AOR = 2.8, (95% CI; 1.1–6.9)], and older than 26 years [AOR = 4.8, (95% CI; 1.5–15.5)] had higher odds of intention to quit khat chewing compared to those who started chewing at age 15 and below. The odds of intention to quit khat chewing among adults who use additional substances (cigarette, hashish, alcohol) daily was 63% less likely than those who use sometimes [AOR = 0.37, (95% CI; 0.16–0.85)]. Adults who had high environmental reevaluation score were about 2 times more likely have the intention to quit chewing than those who had low environmental reevaluation score [AOR = 1.9, (95% CI: 1.1–3.3)]. Khat chewers who had high self-reevaluation score were 3 times more likely have the intention to quit chewing than those who had low self-reevaluation score [AOR = 2.8 (95% CI: 1.6–5.0)]. Participants who had high social liberation score were about 2 times more likely have the intention to stop khat chewing than those with low social liberation score [AOR = 1.8, (95% CI: 1.0–3.1)]. Respondents who had a high khat dependence were 39% less likely to have the intention to stop khat chewing as compared to those low khat dependence [AOR = 0.61, (95%CI: 0.38–0.99)] (See Table 3).

**Table 3:** Multiple logistic regression analysis of factors associated with intention to stop khat chewing among Khat chewers in Gondar city, northwest Ethiopia; 2019

Variable	Category	Intention to stop khat chewing		COR,95%CI	AOR, 95%CI
		Yes	No		
Age at start of khat chewing	≤15	22	32	1	1
	16–20	243	107	2.3 (1.8–2.9)	3.2 (1.5–6.9) *
	21–25	96	34	2.8 (1.9–4.2)	2.8 (1.1–6.9) *
	≥26	58	13	4.4 (2.4–8.1)	4.8 (1.5–15.5) *
Frequency of additional drug use	Sometimes	96	30	1	1
	1–3 days	67	24	2.8 (1.8–4.5)	0.51 (0.2–1.2)
	4–6 days	76	30	2.5 (1.7–3.9)	0.63 (0.26–1.5)
	Day to day	84	53	1.6 (1.1–2.2)	0.37 (0.16–0.8) *
Khat dependency	Low	222	88	1	1
	Medium	20	12	1.7 (0.8–3.4)	0.72 (0.25–2.0)
	High	177	86	2.5 (2.0–2.7)	0.61 (0.38–.9) *
Environmental Reevaluation	Low	87	101	1	1
	Medium	28	10	2.7 (1.3–5.6)	2.0 (.78–5.4)
	High	302	77	4.0 (3.1–5.2)	1.9 (1.1–3.3) *
Self-Reevaluation	Low	94	111	1	1
	Medium	25	12	2.4 (1.1–5.0)	1.6 (0.6–4.2)
	High	298	65	4.6 (3.5–6.0)	2.8 (1.6–5.0) *
Social Liberation	Low	110	104	1	1
	Medium	42	27	1.6 (1.0–2.6)	1.1 (.53–2.3)
	High	265	57	4.7 (3.5–6.2)	1.8 (1.01–3.1) *

*Statistically Significant at *p*-value < 0.05

## Discussion

Despite finding on the prevalence of khat chewing, study reports on intention to stop chewing and quitting attempts aid policymakers and programmers in designing appropriate interventions at different levels. In this study majority of participants (69.3%) had an intention to stop khat chewing within the next 6 months or before. This finding is consistent with a study conducted in Dessie city (Ethiopia) [29]. On the other hand, this finding is higher than studies done in Saudi Arabia (29.5%) [33], male Yemeni adult residents in the UK (46%) [34] and UK (55%) [35]. This could be due to differences in socio-demographic characteristics of the study population. The other reason might be due to difference in the way intention to quit khat chewing was measured. Intention to quit khat chewing was measured by a single question in Saudi Arabia, Yemeni, and UK studies. While in our study intention was measured by using the staging algorithm developed by Prochaska, Diclemente, and colleagues (contemplation and preparation stages) [19].

In the present study, the mean age of initiation of khat chewing was 20.42 (SD ± 4.3) years. This finding is in line with previous studies conducted in Dessie (Ethiopia) [29], Saudi Arabia [33], and the UK [35] but higher than the finding reported among Yemeni adults in the UK [34]. In this study, khat chewers who started chewing at older age had increased odds of intention to quit khat chewing than those who start at a younger age. Adults who started chewing at an earlier age had a prolonged khat chewing experience and higher khat dependence level. As a result, khat chewers who started chewing at a younger age (15 years and below) less likely intend to stop chewing. Likewise, this study also found that intention to stop khat chewing was lower among chewers who had high khat dependence levels. A study done in Saudi Arabia reported that low intention to stop khat chewing is potentially due to high level of khat dependence. This may indicate the gaps in prevention strategies on substance abuse in general and khat use in particular [33].

The present study found that individuals who use additional drugs daily (cigarette, hashish, alcohol) were less likely intend to quit khat chewing as compared to those who use sometimes. Since using one would tempt the use of others concurrent substance use remains as a main barrier of intention to quit and quitting process. In line with this, the systematic review and meta-analysis among university students in Ethiopian reported alcohol drinking and cigarette smoking as a predictor of khat chewing [11]. This finding was consistent with a qualitative study done among khat users and healthcare professionals in London [36]. In contrast, other drug use was not associated with khat use or quitting in the study done among khat quitters in the Jazan area of Saudi Arabia [18].

In TTM the processes of change are the covert and overt activities that people use to progress through stages of changes [22]. The current study revealed that the participant who had high environmental reevaluation, self-reevaluation and social liberation more likely intend to quit khat chewing than their counterparts. Environmental reevaluation is realizing the negative impact of khat chewing on one’s own social and/or physical environment [37, 38]. In our study, adults who had high environmental reevaluation scores had two times more likely have the intention to quit khat chewing compared to those who had a low environmental reevaluation score. Similarly, individuals who had higher social liberation score were two times more likely have the intention to stop khat chewing than those with low social liberation score. Self-liberation is realizing that social norms are changing in the direction of supporting health behavior change [37, 38]. This finding is inconsistent with studies done in Dessie city, which reported medium environmental reevaluation and social liberation score are associated with increased intention to quit khat chewing [29]. Intention to quit does not vary between high, medium and low environmental reevaluation and social liberation process of changes in a study done in Dire Dawa town [30]. The possible reason might be the difference in sociocultural and demographic factors between the study areas.

On the other hand, individuals with high self-reevaluation were 3 times more likely to have the intention to quit khat chewing than those with low self-reevaluation. Self-reevaluation is realizing that behavior change is an important part of one’s identity [37, 38]. This result is in line with the a study done in Dire Dawa town [30] and whereas, contradict with finding in Dessie town [29]. The observed difference might be due to sociocultural variation between the study populations. Other variables in TTM like, consciousness-raising, dramatic relief, self-efficacy, and decisional balance were not significantly associated with intention to quit khat chewing.

### Limitation of the study

There are a few limitations to this study. First, in the TTM the lines between the stages are both stable and open to change. From the stage algorithm of the model precontemplation, contemplation and preparation stages were included in this study and 6 months of period used as criteria for determine intention. Second, since khat chewing behavior is sensitive there may be social desirability that overestimates the intention to quit chewing. We have provided full anonymity to all participants. As such, we believe that the response biases have been kept to a minimum. Being a cross-sectional study may not allow establishing to draw causal relationships among the variables.

## Conclusion

The majority of the khat chewers had intention to stop khat chewing at least within the next 6 months. Age of khat chewing initiation, daily use of additional drugs, khat dependency, environmental reevaluation, self-reevaluation, and social liberation were significant predictors of intention to stop khat chewing. Thus, stage-based interventions should be taken to personalized risk, alter risk perception, clarify values and misconceptions. In addition to intervention to decrease substance abuse, skill training and modeling (who have previously overcome difficult barriers) need to be emphasized to enhance khat chewers’ intention to stop chewing. On the other hand, it is important to assist chewers’ in their quit attempts to increase success rates. Reducing khat chewing habit have an important effect on health and economic development.

## Data Availability

The datasets used in the current study are available from the corresponding author on reasonable request.
